# Can the Chinese study on the normal range of FeNO in children evaluate standardized asthma treatment efficacy in 6- to 12-year-old children?

**DOI:** 10.3389/fped.2023.1189496

**Published:** 2023-09-19

**Authors:** Qiuyan Yang, Chunling Cai, Qingrong Xu, Yuehong Zheng, Aijun Li, Ying Liu, Shufang Li, Yanli Zhang

**Affiliations:** ^1^ Department of Pediatrics, Third Affiliated Hospital of Zhengzhou University, Zhengzhou, China; ^2^ Henan Pediatric Clinical Research Center, Zhengzhou, China; ^3^ Henan Key Laboratory of Child Brain Injury, Zhengzhou, China; ^4^ Institute of Neuroscience of Zhengzhou University, Zhengzhou, China

**Keywords:** normal values of FeNO, pulmonary function parameters, FeNO measurement, repeated-measures ANOVA, therapy

## Abstract

**Objective:**

By examining fractional exhaled nitric oxide (FeNO) levels and performing pulmonary function testing, this study explored whether the multicenter study on the normal range of FeNO in children in China can be used to evaluate standardized treatment efficacy in 6- to 12-year-old children with asthma.

**Methods:**

A total of 115 children aged 6–12 years old who were first diagnosed with asthma and received standardized asthma treatment from April 2018 to July 2022 were selected. According to the FeNO level at the first visit, the subjects were divided into different high- and low-FeNO groups according to the American Thoracic Society (ATS) guidelines and the Chinese multicenter study recommendations. The consistency of the two grouping methods and the differences between the high- and low-FeNO groups were compared after standardized treatment. The grouping method that was the most suitable for children in the cross group was discussed.

**Results:**

(i) There was fair consistency between the Chinese multicenter study recommendations and the ATS guidelines regarding the classification of high- and low-FeNO groups (*Kappa *= 0.338). (ii) Repeated-measures ANOVA showed that the level of improvement in FVC%, FEV1%, FEF25%, FEF50%, and FeNO in the American high- and low-FeNO groups differed with the duration of therapy (*P *< 0.05), however, there was no significant difference between the Chinese groups. (iii) FEV1% and FeNO improved more after treatment in the fixed high-FeNO group than in the cross group (*P* < 0.05).

**Conclusion:**

The Chinese multicenter study on the normal range of FeNO in children in China has a limited role in evaluating standardized asthma treatment efficacy in 6- to 12-year-old children. The ATS guidelines are currently recommended for clinical assessment of asthma treatment efficacy.

## Introduction

Asthma is a chronic inflammatory disease of the airways caused by a combination of inflammatory cells and inflammatory mediators (1). It has a high prevalence in children and shows an increasing trend year by year (2). In asthma patients, the expression of inducible nitric oxide synthase is increased in airway epithelial cells, which increases the concentration of nitric oxide in exhaled air (3). As it is a noninvasive and simple measurement for assessing airway inflammation, the FeNO test is widely used in asthma patients and easily performed starting from 5 to 6 years of age (4, 5).

The Global Initiative for Asthma (GINA) states that the FeNO test can be used to support the diagnosis of asthma and to monitor the response to anti-inflammatory therapy with inhaled corticosteroids (ICSs) (6). The FeNO test is a first-line test for the diagnosis of asthma in children (7), and the ATS guidelines indicate that it supports a diagnosis of asthma when objective evidence is available (8). A systematic evaluation showed that FeNO has considerable accuracy in the diagnosis of asthma, with a diagnostic specificity of 82% (9). However, its role in instituting asthma treatment protocols is limited (10). It is an important indicator in asthma management (11). In asthma therapy, FeNO can be used to assess treatment adherence in asthma patients and can predict patient response to inhaled glucocorticoids (ICSs) (12) and different biologics (5).

FeNO levels are influenced by many factors, of which age is one of the most important (5, 13–15). The ATS guidelines, which are currently the most widely used in clinical practice, also state that age is a factor that affects FeNO levels in children under 12 years of age (8). FeNO levels are positively correlated with age (16). FeNO levels can be used to evaluate treatment response (5, 8, 12), but more detailed (age-specific) assessment criteria for children are lacking. The ATS guidelines do not define FeNO levels for children according to age; however, they recommend the FeNO cutoff value of 20 ppb for children (8).

A 2020 multicenter study of normal FeNO values in school-aged children and adolescents aged 6–18 years in China (referred to as the Chinese multicenter study below) defined normal FeNO values according to age. For children aged 12–18 years, a cutoff point of 16 ppb is recommended, and for children under 12 years, the FeNO cutoff point is reduced by 1 ppb for each year of age (17). As with the ATS guidelines, the Chinese multicenter study also showed that age affected FeNO levels in children under 12 years old. However, compared with the ATS guidelines, the Chinese multicenter study provided more detailed criteria on normal FeNO values for 6- to 12-year-old children Whether the Chinese multicenter study recommendations can be used to evaluate standardized asthma treatment efficacy in 6–12 years old children needs further investigation.

In this study, we focused on the role of FeNO in the assessment of asthma treatment efficacy. To investigate whether the normal range of FeNO in Chinese children can be used to evaluate standardized asthma treatment efficacy in 6- to 12-year-old children. This study employed the FeNO cutoff values from the ATS guidelines and the Chinese multicenter study, grouped the study subjects according to the different cutoff values, and compared the differences in pulmonary function test results between the high- and low-FeNO groups before and after treatment.

## Methods

### Study population

School-aged children aged 6–12 years who were first diagnosed with asthma in the outpatient clinic from April 2018 to July 2022 were included in this study and received standardized asthma treatment. The children were followed up regularly at the outpatient clinic 1 month and 3 months after the initial diagnosis. The children were free of respiratory infections for 4 weeks before the initial diagnosis. In addition, their asthma symptoms were well controlled [Asthma Control Questionnaire (ACQ) score ≤1.5], and no changes in the treatment regimen were made during the treatment period. The diagnostic criteria, standardized treatment, and asthma management were based on the Global Asthma Initiative published in 2020 (6). Children with other lung diseases that may affect pulmonary function testing, such as congenital malformations of airway development, were excluded. Children who failed to cooperate with treatment and follow-up in a timely manner were also excluded. Children whose comorbidities (e.g., allergic rhinitis, nasosinusitis) were not well controlled during the treatment were excluded.

**Figure 1:**
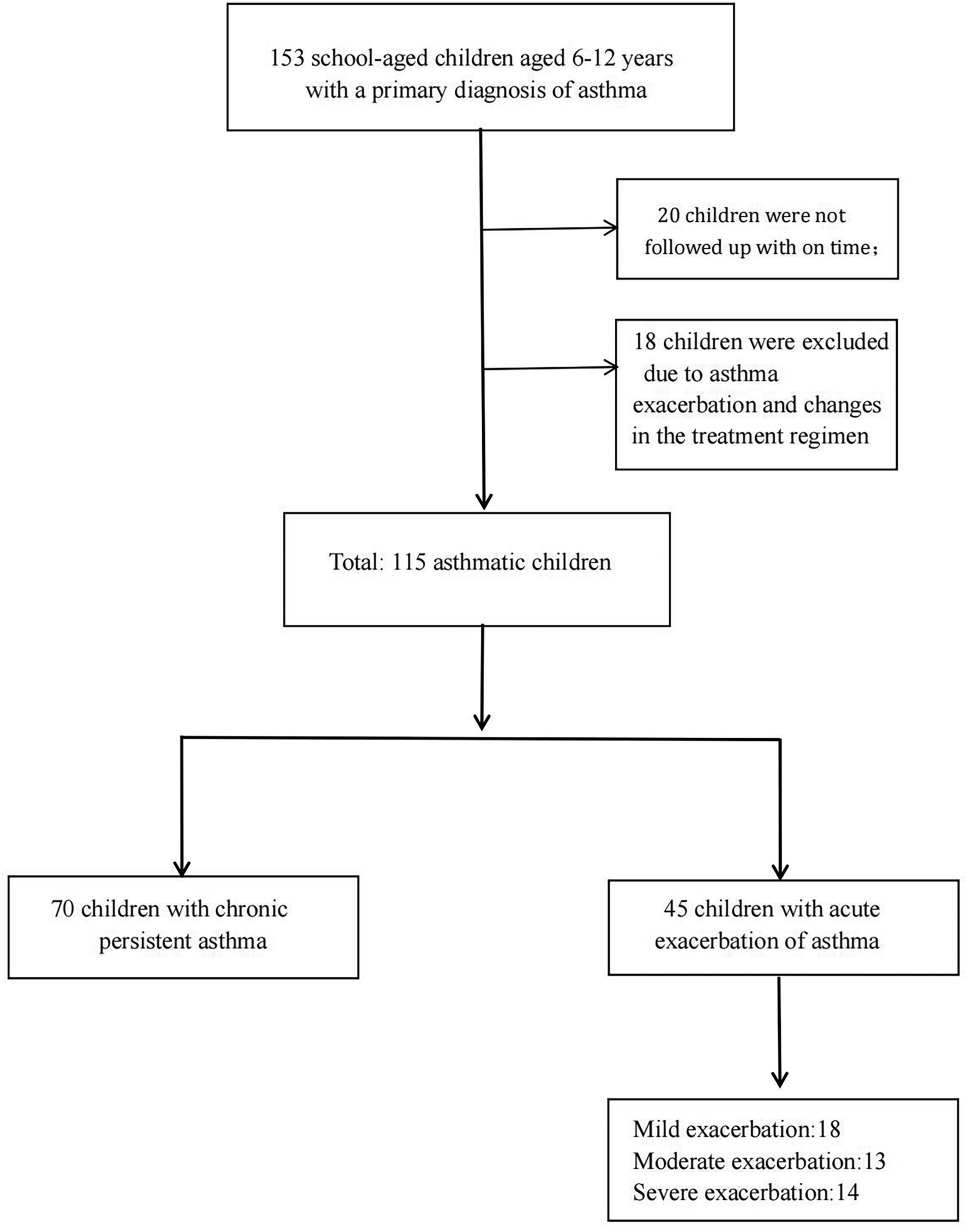
Flow chart of patient inclusion.

A total of 153 school-aged children aged 6–12 years with a primary diagnosis of asthma were included in this study. Twenty children were not followed up in a timely manner, and eighteen children were excluded due to asthma exacerbation and changes in the treatment regimen. There were 70 children with chronic persistent asthma and 45 children with acute exacerbation of asthma. Among the children in the acute exacerbation period, a total of 18, 13, and 14 children had mild, moderate, and severe exacerbations, respectively. A total of 115 children were included in this study (Figure 1). All 115 subjects had asthma symptoms most days, or they woke up one or more times per week because of asthma before the first visit. At the first visit, all subjects underwent pulmonary function testing and FeNO measurements. Then, according to the disease status of the children and the corresponding examination results, a standardized asthma treatment plan was developed by the pediatrician in accordance with the guidelines (6). Each child was given combined treatment with an ICS and a long-acting beta-agonist [budesonide-formoterol powder (80 µg/4.5 µg/inhalation) once in the morning and once at night]. Guidance on the use of the medication was also given to ensure that the medication was inhaled correctly. Atopy was defined as sensitization to at least 1 common food or respiratory allergen (such as meat, wheat, mites, molds, pets, and plants). Allergen was measured by allergen-specific skin prick test reactivity or IgE sensitization. One month and three months after the initial diagnosis, the subjects underwent routine follow-up at the hospital outpatient clinic. Their medicine was administered correctly and regularly during therapy. Pulmonary function testing and FeNO measurements were repeated at the follow-up visits. A pediatrician also assessed each child's disease condition according to their symptoms and completed the ACQ questionnaire (18).

### Study design

The purpose of this study was to explore whether the normal range of FeNO among children aged 6–18 years old in China could be used to evaluate standardized asthma treatment efficacy in 6- to 12-year-old children. According to the FeNO level at the first visit, the subjects were divided into different high- and low-FeNO groups according to the ATS guidelines and the FeNO cutoff values recommended by the Chinese multicenter study. The consistency of the two grouping methods was examined, and the differences between the high- and low-FeNO groups, grouped according to different grouping methods, after standardized treatment were compared to determine which grouping method was most appropriate for children in the cross group.

According to the FeNO level at the first visit, the subjects were divided into the Chinese high-FeNO group (≥ the normal FeNO value at the same age level) and the Chinese low-FeNO group (< the normal FeNO value at the same age level) according to the normal value recommended by the Chinese multicenter study (17). At the same time, the children were divided into the American high-FeNO group (≥20 ppb) and the American low-FeNO group (<20 ppb) based on the ATS guidelines (8). The consistency of the two grouping methods was determined, and the differences in pulmonary function parameters and FeNO levels between the Chinese high- and low-FeNO groups and the American high- and low-FeNO groups were compared. The differences in lung function parameters and FeNO between the high- and low-FeNO groups classified according to the two criteria mentioned above were analyzed after standardized treatment. In addition, the children were divided into the fixed high-FeNO group (in both the Chinese and American high-FeNO groups), the fixed low-FeNO group (in both the Chinese and American low-FeNO groups), and the cross group (in the Chinese high-FeNO group and the American low-FeNO group). The differences in pulmonary function parameters and FeNO changes between the cross group and the fixed high- and low-FeNO groups were compared before and after treatment to investigate the changes in the pulmonary function of children in the cross group after standardized treatment. Subsequently, it was clear which grouping method was more appropriate for children in the cross group.

### FeNO measurement

FeNO measurements were performed according to the guidelines for FeNO measurement developed by the American Thoracic Society/European Respiratory Society (ATS/ERS) (19) and the manufacturer's instructions. Before pulmonary function testing, FeNO measurements were performed using a nitric oxide analyzer (SV-02, Wuxi Shangwo, China). The study subjects were instructed to abstain from nitrite-containing foods for 2 h before the test and to abstain from food, water, strenuous exercise and passive smoking for 1 h before the test. To ensure the homogeneity of the test results, the measurements were all performed by the same professionals who had received standardized training. During the measurement, the children exhaled smoothly and slowly at 50 ml/s for 10 s. The unit of the test results is expressed in parts per billion (ppb).

### Pulmonary function testing

A certified specialist technologist performed rigorous pulmonary function testing using a pulmonary function analyzer (Type Master Screen IOS, Cardinal Health Germany 234 GmbH Leibnizstrasse 7 D-97204 Hochberg, made in Germany) according to the guidelines (20). The examined pulmonary function parameters included forced vital capacity (FVC), forced expiratory volume in one second (FEV1), the ratio of the forced expiratory volume in 1 s to the forced vital capacity (FEV1/FVC), peak expiratory flow (PEF), forced expiratory flow at 75% of vital capacity (FEF25), forced expiratory flow at 50% of vital capacity (FEF50) and forced expiratory flow at 25% of vital capacity (FEF75). The pulmonary function parameters are expressed as the percentage ratio of the measured value to the predicted value. Each subject performed at least 3 trials (no more than 8), and the best outcome was selected for data analysis.

### Sample size

The sample size was calculated using G*Power software version 3.1.9.7. This study used ANOVA to analyze the time, group and interaction effects of the interventions. A minimum sample of 102 subjects was used, keeping the minimal significance (α) and statistical power (1-β) at 0.05 and 0.95, respectively.

### Statistical analysis

In this study, IBM SPSS statistical software (SPSS 26.0, Chicago, Illinois) was used for data analysis. The Kappa consistency test was used to analyze the consistency between the Chinese multicenter study recommendations and the ATS guidelines regarding the grouping of high and low FeNO levels. The measurement data are expressed as the mean ± standard deviation (SD) or median (interquartile range) [M (P_25_, P_75_)]. When analyzing differences in continuous data between two groups, either the independent samples *t*-test or the Mann–Whitney *U*-test was employed, depending on the data. Enumeration data are expressed as a percentage (%), and the differences in categorical data between groups were analyzed using the chi-square (χ^2^) test. The repeated measurement data were analyzed with repeated-measures ANOVA. A Bonferroni correction was used to correct for multiple comparisons. *P *< 0.05 indicated that the difference was statistically significant.

## Results

### Consistency of the Chinese multicenter study recommendations and the ATS guidelines in high and low FeNO grouping

The 115 study subjects were grouped according to the Chinese multicenter study recommendations and the ATS guideline criteria. The results of the consistency test showed that the Chinese multicenter study recommendations and the ATS guidelines were fairly consistent in terms of high and low FeNO level grouping (*Kappa *= 0.338, Table 1).

**Table 1:** Consistency of the Chinese multicenter study recommendations and the ATS guidelines in grouping.

The Chinese multicenter study	The ATS guidelines		*Kappa* value	*P*-value
	The American low group	The American high group		
The Chinese low group	30	0	0.338	<0.001
The Chinese high group	43	42		

The consistency level represented by Kappa coefficient: *Kappa *≤ 0.2, slight; 0.2 < *Kappa *≤ 0.4, fair; 0.4 < *Kappa *≤ 0.6, moderate; 0.6 < *Kappa *≤ 0.8, substantial; 0.8 < *Kappa*, almost perfect.

### Demographic characteristics of each group at the first visit (baseline)

Pairwise comparisons were made between the Chinese high and low groups and the American high- and low-FeNO groups. There were no significant differences in demographics or lung function parameters between the high- and low-FeNO groups (*P* > 0.05, Table 2), except for the baseline FeNO level (*P* < 0.001, Table 2).

**Table 2:** Demographic characteristics of the Chinese high and low groups and the American high and low groups at the first visit.

Variables	All (*N* = 115)	The Chinese multicenter study		*P*-value	The ATS guidelines		*P*-value
		The Chinese low group (*N* = 30)	The Chinese highgroup (*N* = 85)		The American lowgroup (*N* = 73)	The American highgroup (*N* = 42)	
Sex, male	83 (72.2)	23 (76.7)	60 (70.6)	0.523	52 (71.2)	31 (73.8)	0.767
Age, year	7.00 (7.00, 9.00)	7.50 (7.00, 9.00)	7.00 (7.00, 9.00)	0.804	7.00 (6.00, 8.00)	8.00 (7.00, 9.00)	0.067
Height, cm	127.75 ± 9.53	127.83 ± 11.02	127.72 ± 9.01	0.957	126.80 (120.25, 133.65)	128.6 (120.38, 134.63)	0.638
Weight, kg	26.0 (21.90, 30.90)	25.85 (21.83, 31.60)	26.00 (22.35, 30.80)	0.868	26.00 (22.10, 30.65)	26.40 (21.25, 32.05)	0.912
BMI, kg/m^2^	16.00 (14.70, 17.60)	15.90 (14.70, 18.18)	16.30 (14.80, 17.50)	0.962	16.30 (14.80, 17.70)	15.95 (14.40, 17.03)	0.571
FeNO, ppb	17.00 (10.00, 26.00)	8.00 (7.00, 10.00)	19.00 (15.00, 29.50)	<0.001*	12.00 (8.50, 16.00)	29.50 (25.00, 48.50)	<0.001*
FVC% predicted	98.10 (90.90, 103.10)	96.25 (89.35, 107.83)	98.30 (91.25, 103.10)	0.954	97.52 ± 11.19	95.76 ± 12.01	0.430
FEV1% predicted	95.10 (86.90, 99.50)	92.55 (81.03, 98.70)	95.30 (87.65, 100.30)	0.299	95.30 (85.90, 100.25)	92.60 (87.78, 98.53)	0.617
FEV1/FVC% predicted	91.48 ± 8.68	90.33 ± 7.15	91.88 ± 9.17	0.403	92.33 ± 8.72	88.99 ± 8.52	0.166
PEF% predicted	82.72 ± 15.59	81.71 ± 17.77	83.08 ± 14.85	0.681	83.24 ± 15.91	81.82 ± 15.16	0.641
FEF25% predicted	78.28 ± 16.73	74.61 ± 18.86	79.58 ± 15.83	0.163	78.30 ± 16.61	78.24 ± 17.13	0.985
FEF50% predicted	66.75 ± 16.81	62.82 ± 16.49	68.13 ± 16.80	0.137	67.62 ± 15.92	65.23 ± 18.35	0.467
FEF75% predicted	48.38 ± 16.10	43.89 ± 14.50	49.96 ± 16.41	0.075	48.48 ± 16.11	48.19 ± 16.27	0.926

Data are shown as mean ± standard deviation, [*M* (*P*_25_, *P*_75_)] or frequency (percentage).

BMI, body mass index; FeNO, fraction of exhaled nitric oxide; ICS, inhaled corticosteroid; LABA, long-acting β_2_-agonists.

* *P* < 0.05.

At the first visit, the level of FeNO in the cross group was significantly higher than that in the fixed low-FeNO group (*P *< 0.001, Table 3), but there were no significant differences in other variables (*P *> 0.05, Table 3). The cross group had significantly lower FeNO levels than the fixed high-FeNO group, and the difference was statistically significant (*P *< 0.001, Table 3). In contrast, the differences between the other variables were not statistically significant (*P *> 0.05, Table 3).

**Table 3:** Demographic characteristics of the cross group and the fixed high and low groups at the first visit.

Variables	The cross group (*N* = 43)	The fixed low group (*N* = 30)	The fixed high group (*N* = 42)	The cross group vs. the fixed low group *P*-value	The cross group vs. the fixed high group *P-*value
Sex, male	29 (67.4)	23 (76.7)	31 (73.8)	0.392	0.519
Age, year	7.00 (6.00,8.00)	7.50 (7.00,9.00)	8.00 (7.00,9.00)	0.519	0.054
Height, cm	127.58 ± 9.01	127.83 ± 11.02	127.86 ± 9.12	0.916	0.886
Weight, kg	27.00 ± 6.10	25.85 (21.83,31.60)	26.99 ± 6.67	0.814	0.997
BMI, kg/m^2^	16.40 (15.00, 17.60)	15.90 (14.70, 18.18)	15.95 (14.40, 17.03)	0.784	0.565
FeNO, ppb	15.00 (13.00, 18.00)	8.00 (7.00, 10.00)	29.50 (25.00, 48.50)	<0.001*	<0.001*
FVC% predicted	97.96 ± 9.90	96.88 ± 12.98	95.76 ± 12.01	0.688	0.357
FEV1% predicted	95.43 ± 11.39	90.09 ± 14.36	92.60 (87.78,98.52)	0.081	0.298
FEV1/FVC% predicted	93.73 ± 9.50	90.33 ± 7.15	89.99 ± 8.52	0.102	0.060
PEF% predicted	84.31 ± 14.60	81.71 ± 17.77	81.82 ± 15.16	0.496	0.444
FEF25% predicted	80.88 ± 14.52	74.61 ± 18.86	78.24 ± 17.13	0.132	0.446
FEF50% predicted	70.96 ± 14.80	62.82 ± 16.49	65.23 ± 18.35	0.051	0.117
FEF75% predicted	51.69 ± 16.55	43.89 ± 14.50	48.19 ± 16.27	0.061	0.339

Data are shown as mean ± standard deviation, [*M* (*P*_25_, *P*_75_)] or frequency (percentage).

BMI, body mass index; FeNO, fraction of exhaled nitric oxide; ICS, inhaled corticosteroid; LABA, long-acting β_2_-agonists.

* *P* < 0.05.

### Results for the time ▪ group interaction in repeated-measures ANOVA

**Figure 2:**
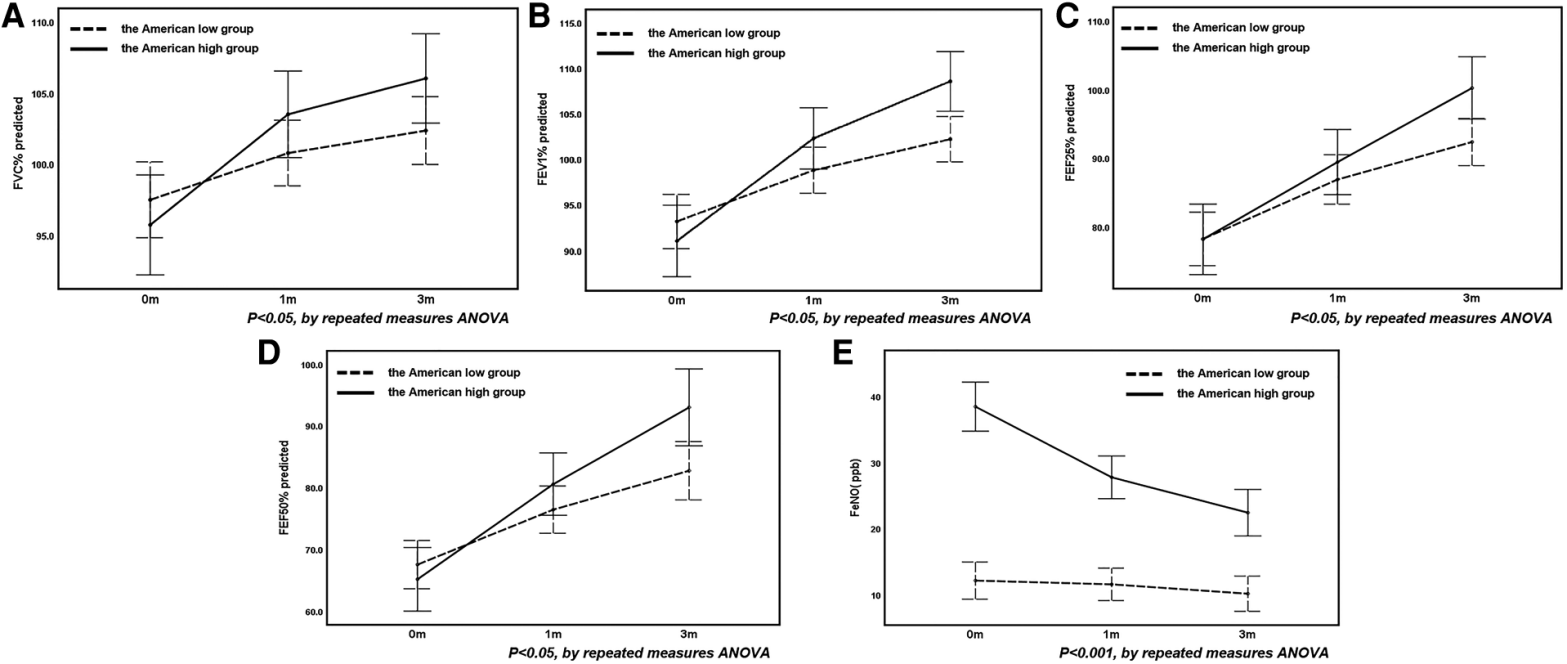
The changes in FVC% (**A**), FEV1% (**B**), FEF25% (**C**), FEF50% (**D**) and FeNO (**E**) before and after treatment in the American high and low groups.

There was no significant difference in the improvement of pulmonary function parameter between the Chinese high- and low-FeNO groups as the duration of treatment increased (*P *> 0.05, Table 4). However, the level of improvement in FVC%, FEV1%, FEF25%, FEF50% and FENO in the American high- and low-FeNO groups differed with the duration of therapy (*P *< 0.05, Table 4). Specifically, the improvements in FVC%, FEV1%, FEF25%, FEF50% and FeNO were greater in the American high-FeNO group than in the American low-FeNO group (Figure 2).

**Table 4:** Results for the time ▪ group interaction in repeated-measures ANOVA.

Variables	The Chinese high group vs. the Chinese low group	The American high group vs. the American low group	The cross group vs. the fixed low group	The cross group vs. the fixed high group
FVC% predicted	0.096	0.029*	0.303	0.111
FEV1% predicted	0.222	0.002*	0.933	0.010*
FEV1/FVC% predicted	0.904	0.747	0.817	0.677
PEF% predicted	0.058	0.121	0.110	0.239
FEF25% predicted	0.203	0.017*	0.575	0.064
FEF50% predicted	0.514	0.025*	0.996	0.079
FEF75% predicted	0.585	0.132	0.840	0.256
FeNO	0.051	<0.001*	0.624	<0.001*

* *P* < 0.05.

**Figure 3:**
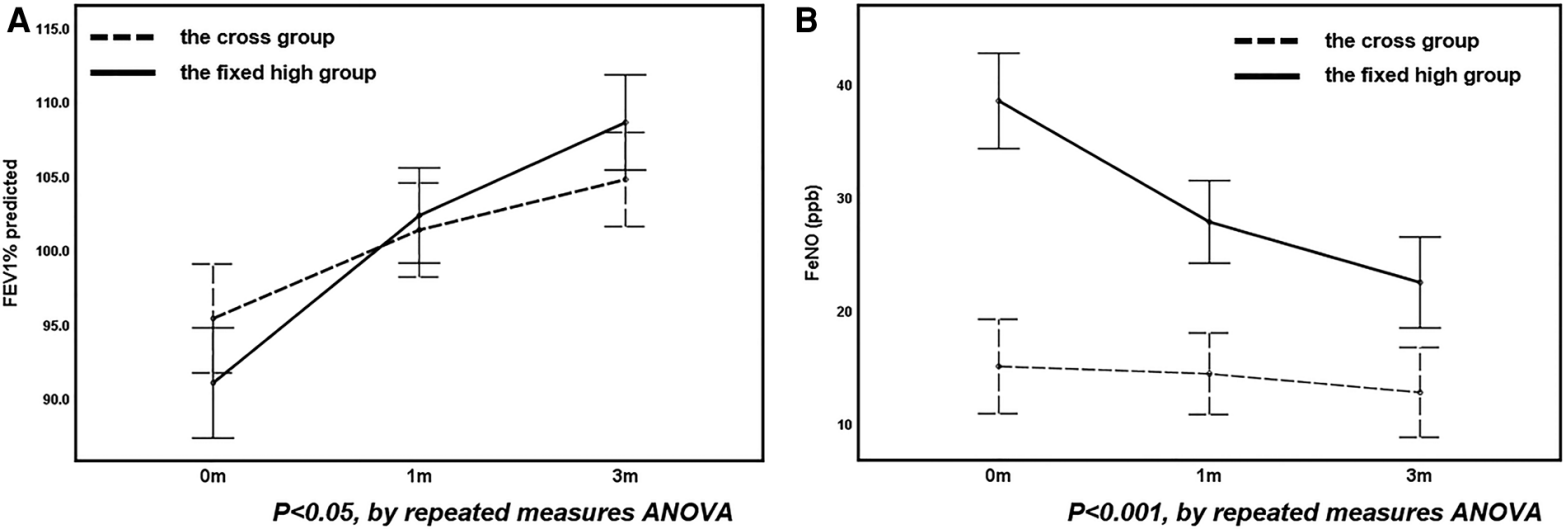
The change in FEV1% (**A**) and FeNO (**B**) before and after treatment in the fixed high group compared with the cross group.

When comparing the cross group with the fixed high- and low-FeNO groups, no differences in the level of improvement in any pulmonary function parameter were observed between the cross and fixed low-FeNO groups as therapy progressed (*P* > 0.05, Table 4). However, the improvements in FEV1% and FeNO were greater in the fixed high-FeNO group than in the cross group (Figure 3).

### Analysis of differences in FVC%, FEV1%, FEF25% and FEF50% before and after treatment in the American high- and low-FeNO groups

FVC%, FEV1%, FEF25% and FEF50% improved gradually after treatment in both the American high- and low-FeNO groups (*P *< 0.001, Table 5). The degree of change in FVC%, FEV1%, FEF25% and FEF50% differed between the American high- and low-FeNO groups after treatment (*P *= 0.029, *P *= 0.002, *P *= 0.017, *P *= 0.025, respectively, Table 5), and the improvement was greater in the American high-FeNO group (Figures 2A–D). However, there was no significant difference in FVC%, FEV1%, FEF25% or FEF50% between the two groups (*P *= 0.395, *P *= 0.180, *P *= 0.176, *P *= 0.151, respectively, Table 5).

**Table 5:** Analysis of differences in FVC%, FEV1% and feNO before and after treatment in the American high and low groups.

Variable	Group	Visit 1 (0 month)	Visit 2 (1 month)	Visit 3 (3 month)	Time *P*-value	Group *P*-value	Interaction/time·group *P*-value
FVC% predicted	L	97.52 ± 1.35	100.81 ± 1.17*	102.39 ± 1.20^*†^	<0.001	0.395	0.029
	H	95.76 ± 1.77	103.53 ± 1.54*	106.06 ± 1.59^*†^			
FEV1% predicted	L	93.24 ± 1.50	98.87 ± 1.28*	102.29 ± 1.26^*†^	<0.001	0.180	0.002
	H	91.09 ± 1.98	102.38 ± 1.69*	108.65 ± 1.66^*†^			
FEF25% predicted	L	78.30 ± 1.97	86.96 ± 1.81*	92.42 ± 1.74^*†^	<0.001	0.176	0.017
	H	78.24 ± 2.59	89.49 ± 2.39*	100.29 ± 2.29^*†^			
FEF50% predicted	L	67.62 ± 1.97	76.51 ± 1.93*	82.81 ± 2.39^*†^	<0.001	0.151	0.025
	H	65.23 ± 2.59	80.64 ± 2.55*	93.06 ± 3.14^*†^			
FeNO	L	12.19 ± 1.42	11.62 ± 1.24	10.19 ± 1.35	<0.001	<0.001	<0.001
	H	38.55 ± 1.87	27.83 ± 1.63*	22.48 ± 1.78^*†^			

Values are mean ± SD.

L, the American low group; H, the American high group.

* *P* < 0.05 compared with baseline.

^†^ *P* < 0.05 compared with the preceding phase.

Compared to the first visit, there were significant increases in FVC%, FEV1%, FEF25% and FEF50% in the American high- and low-FeNO groups after 1 month of treatment (*P *< 0.05, Table 5). In addition, after 3 months of treatment, there were significant increases in FVC%, FEV1%, FEF25% and FEF50% in both groups compared to the values from the initial consultation and after 1 month of therapy (*P *< 0.05, Table 5).

### Analysis of the difference in FeNO levels before and after treatment in the American high- and low-FeNO groups

FeNO levels decreased after treatment in both groups (*P *< 0.001, Table 5). The decrease in FeNO levels after therapy was different between the two groups (*P *< 0.001, Table 5), with a greater decrease in the American high-FeNO group (Figure 2E). Moreover, there was a significant difference in FeNO levels between the two groups after treatment (*P *< 0.001, Table 5).

In the American high-FeNO group, FeNO levels were significantly lower after 1 month and 3 months of treatment than at the first visit (*P *< 0.05, Table 5) and were significantly lower after 3 months of treatment than after 1 month of treatment (*P *< 0.05, Table 5). However, no significant difference was found among time points in the American low-FeNO group (*P *> 0.05, Table 5).

## Discussion

FeNO measurements have been suggested to be of great clinical utility as a form of noninvasive testing for respiratory diseases, particularly asthma (21). Research has shown that FeNO measurement can assist in diagnosing asthma in children (5, 7, 9, 22, 23), evaluating treatment efficacy (5, 24), and managing asthma (5, 24, 25). The influence of age on FeNO levels has been demonstrated in recent years (5, 13–15), but more detailed reference standards for clinical use are currently lacking. FeNO levels increase gradually with age (16, 26). The reason for this may be that the total surface area of the airway mucosa available for NO diffusion increases with age (27). Therefore, older individuals have higher levels of FeNO at the same expiratory flow rate. The guidelines recommend that age is an important factor for children under 12 years of age (8). However, there is a lack of more detailed clinical references for FeNO levels in children with asthma aged from 6 to 12 years. A Chinese multicenter study defined FeNO cutoff values for every year of age, but no studies have confirmed the applicability of these values in the application of standardized asthma treatment for school-aged children. In this study, using the Chinese multicenter study FeNO level criteria, the subjects were divided into high- and low-FeNO groups at the first visit, and the differences between the two groups before and after standardized asthma treatment were analyzed. Moreover, the ATS guidelines were used for comparison, and we conclude that the Chinese multicenter study recommendations have limited value in evaluating standardized asthma treatment efficacy in 6- to 12-year-old children.

This study found fair consistency between the ATS guidelines and the Chinese multicenter study recommendations in terms of high- and low-FeNO grouping. We speculate that the difference may be related to the selection of the reference population. In addition to age, FeNO levels are positively correlated with height and body mass index (BMI) (17), and some studies have indicated that BMI increases with economic growth (28). Studies in China and the United States have shown that FeNO levels increase with increasing BMI (17, 29, 30). Studies in China have shown an increase in obesity and overweight rates as the economy grows (31). The reference population for the Chinese multicenter study was from China, a developing country, while those for the ATS guidelines were from developed countries such as New Zealand and the USA. The growth and nutritional status of children in developed countries are markedly higher than those in developing countries. Therefore, the FeNO detection rates were low in the high-FeNO group and high in the low-FeNO group based on the ATS guidelines. Ethnicity could also be a potential factor (32), and the effects of the environment cannot be ignored. The mean value of FeNO for healthy Asian Canadian children aged 9.1–12.9 years was 22.8 ppb (33), which was much higher than that of Chinese children in the same age group (17).

Additionally, this study found no difference in the level of improvement in any pulmonary function parameter between the Chinese high- and low-FeNO groups after treatment. In contrast, the improvement in FVC%, FEV1%, FEF25%, FEF50% and FeNO after treatment was greater in the American high-FeNO group than in the American low-FeNO group. This difference was considered related to the cross-group population. Further analysis revealed that the levels of change in all pulmonary function parameters and FeNO after treatment in the cross group were similar to those in the fixed low-FeNO group. Thus, the ATS guideline grouping approach was more appropriate for the cross group. This result may be because the Chinese multicenter study excluded children with atopy or allergies, whereas 87.8% of the children in this study had allergies. It has been shown that FeNO levels are higher in patients with atopy (34). Therefore, the FeNO levels of the subjects in this study were higher than the standards of the Chinese multicenter study for participants of the same age. It is also possible that there were different responses to ICS treatment between individuals with high and low FeNO levels. Asthma patients with high FeNO levels have better improvements in asthma status after ICS treatment. However, patients with low FeNO levels respond poorly to ICS treatment (8, 35). This conclusion is consistent with the results of this study.

Previous studies have shown (36, 37) that patients with high FeNO levels have a poor prognosis, given the risk of decreased FEV1. In contrast to previous studies, FVC% and FEV1% improved after treatment in both the American high- and low-FeNO groups in this study. This result was considered in terms of the study population and the length of the study. Previous studies included adults, and the period of study was longer. However, the subjects in this study were 6- to 12-year-old children who were newly diagnosed with asthma, mostly in the acute phase of the disease, and all children showed improvements in pulmonary function testing outcomes with short-term treatment. Moreover, this study found that the improvement in FeNO levels was significantly lower in the American high-FeNO group after 3 months of treatment compared to the first visit, while there was no difference in the American low-FeNO group. Endo et al. (38) found the same result.

In clinical work, physicians design treatment regimens for patients by referring to their FeNO levels (25, 39, 40). Some studies have confirmed the clinical utility of the FeNO test in diagnosis (23) or customized treatment (41), but its functions are still debated. The diagnosis and treatment of children with asthma are currently based on signs and symptoms (6, 10). A study confirmed that FeNO levels could be used as a predictor of improved asthma control after starting inhaled steroid therapy (35). FeNO-based asthma management can reduce the asthma exacerbation rate in children compared to standardized treatment (42) and reduce the cost of treatment (4).

Age is a factor that affects FeNO values in children under 12 years of age (8). Additionally, the relationship between FeNO levels and airway hyperresponsiveness (AHR) varies by age. A decrease in AHR was significantly associated with elevated FeNO levels in children aged 5–11 years, but this finding was not found in adolescents older than 12 years (15). However, previous studies have confirmed that age affects FeNO levels in children under 12 years old (8, 17). There is a lack of age-based FeNO criteria for the application of standardized treatment in 6–12-year-old children with asthma, which is a problem for clinical practice. It is especially important to accurately divide children into high- and low-FeNO groups. Therefore, this study provides a reference for the assessment of the efficacy of asthma treatment in children. This study used an innovative approach to explore the value of the Chinese multicenter study recommendations for the application of standardized treatment in 6- to 12-year-old children with asthma. However, regrettably, based on the current sample data, this study showed that the role of the Chinese multicenter study recommendations in the application of standardized treatment efficacy in 6- to 12-year-old children with asthma is limited. The results failed to provide precise guidance for evaluating clinical treatment efficacy. The role of the FeNO test in asthma management has been widely recognized. As the understanding of FeNO is deepening, it is believed that new approaches to its application in asthma management will be discovered.

This research offers a distinctive viewpoint by delving into the relevance of the Chinese multicenter study concerning the standard range of FeNO in children, specifically when evaluating the efficacy of standardized asthma treatments. Furthermore, our research juxtaposes the ATS guidelines with the recommendations of the Chinese multicenter study, shedding light on the congruencies and disparities between various international and regional standards. Notably, our conclusions stem from a dynamic evaluation of post-treatment pulmonary ventilatory function changes, analyzed using repeated-measures ANOVA. These findings underscore the indispensable role of the ATS guidelines in the clinical evaluation of asthma treatment effectiveness.

There are several limitations to this study. First, the size of the sample was small and failed to include other centers for a multicenter study. Second, the time span between the before and after treatment studies was short. Third, this study only examined pulmonary function test parameters and did not use other indicators for a more comprehensive and long-term assessment of changes before and after standardized treatment in school-aged children with asthma. Our previous study confirmed the value of the FeNO test in the management of asthma in school-aged children (23). In the next phase of this work, we will increase the sample size, examine more inflammatory indicators, and conduct a longer study to examine FeNO levels in 6- to 12-year-old children with asthma at different ages, assess the value of FeNO levels at different ages in the application of standardized asthma treatment, and provide a reference for the precise treatment of children with asthma.

## Conclusion

In summary, classification with the ATS guidelines was more suitable for children in the cross group. The Chinese multicenter study on the normal range of FeNO in children in China has a limited role in evaluating standardized asthma treatment efficacy in 6- to 12-year-old children. The ATS guidelines are currently recommended for the clinical assessment of asthma treatment efficacy.

## Data Availability

The datasets presented in this study can be found in online repositories. The names of the repository/repositories and accession number(s) can be found below: https://doi.org/10.6084/m9.figshare.22298893.v1.
